# A PROTEOMICS-BASED MEASURE OF ACCELERATING AGING IS CORRELATED WITH THE BRAIN AGE GAP IN THE ARIC STUDY

**DOI:** 10.1093/geroni/igae098.2303

**Published:** 2024-12-31

**Authors:** Ramon Casanova, Keenan Walker, Lingyi Lu, Stephen Kritchevsky, Timothy Hughes, Lynne Wagenknecht

**Affiliations:** Wake Forest University School of Medicine, Winston-Salem, North Carolina, United States; National Institute on Aging, Baltimore, Maryland, United States; Wake Forest University School of Medicine, Winston-Salem, North Carolina, United States; Wake Forest University School of Medicine, Wake Forest University School of Medicine, North Carolina, United States; Wake Forest University School of Medicine, Winston-Salem, North Carolina, United States; Wake Forest University School of Medicine, Winston-Salem, North Carolina, United States

## Abstract

Proteomics clocks can be used to estimate chronologic age or predict age-related outcomes (e.g., GrimAge or AgeAccelGrim and mortality). Analogous to AgeAccelGrim, we developed a proteomic predictor in the Atherosclerosis Risk in Communities (ARIC) Study, and present correlations between this measure and an MRI-derived measure of advanced brain age (brain age gap) and other clinical parameters. We used data from 1447 ARIC participants with both brain MRIs and proteins levels available. The data was partitioned into training and testing datasets (75%-25%). We fitted a Cox regression elastic net model to predict all-cause mortality based on all proteins available in the SOMAscan aptamer panel (N = 4877), age and sex. The linear combination of variables was transformed to match mean age and variance of the training dataset. Finally, the age was regressed out to produce the proteomic measure. The testing dataset was used to estimate Spearman correlations. In total 33 proteins were selected by the model including R-sponding-4, GDF-15, ANGPT2 and PCYOX1L with larger (positives) coefficients while F7, SET and CNDP1 were the ones with smaller (negative) coefficients. The proteomic age measure was correlated with the brain age gap (0.26 p< 0.001), hypertension (0.25 p< 0.001), diabetes (0.18 p< 0.001), gait speed (0.24 p< 0.001), total cholesterol (0.25 p< 0.001) but not correlated with fasting glucose, grip strength or MRI-derived temporal meta-ROI. These results here link brain and body indices of accelerated aging independently of chronological age. Further investigation of the interrelationships between brain and proteomics aging measures is warranted.

